# Antibiotic Resistance in *Helicobacter pylori* Isolates from Northwestern and Central Romania Detected by Culture-Based and PCR-Based Methods

**DOI:** 10.3390/antibiotics12121672

**Published:** 2023-11-28

**Authors:** Carmen Costache, Horațiu Alexandru Colosi, Simona Grad, Anamaria Ioana Paștiu, Mariela Militaru, Anca Paula Hădărean, Dan Alexandru Țoc, Vlad Sever Neculicioiu, Alina Mihaela Baciu, Razvan Vlad Opris, Dan Lucian Dumitrașcu, Ioana Alina Colosi

**Affiliations:** 1 Department of Molecular Sciences, Division of Microbiology, Iuliu Hațieganu University of Medicine and Pharmacy, 400349 Cluj-Napoca, Romania; anca.costache@umfcluj.ro (C.C.); baciu.alina.mihaela@elearn.umfcluj.ro (A.M.B.);; 2 Cluj County Emergency Hospital, 400000 Cluj-Napoca, Romania; costinsimona_m@elearn.umfcluj.ro (S.G.);; 3 Department of Medical Education, Division of Medical Informatics and Biostatistics, Iuliu Hațieganu University of Medicine and Pharmacy, 400349 Cluj-Napoca, Romania; 4 Department of Internal Medicine, 2nd Medical Clinic, Iuliu Hațieganu University of Medicine and Pharmacy, 400000 Cluj-Napoca, Romania; 5 Department of Genetics and Hereditary Diseases, Faculty of Veterinary Medicine, University of Agricultural Sciences and Veterinary Medicine Cluj-Napoca, 400372 Cluj-Napoca, Romania; 6 Department of Molecular Sciences, Division of Medical Genetics, Iuliu Hatieganu University of Medicine and Pharmacy, 400349 Cluj-Napoca, Romania; sanda.militaru@umfcluj.ro; 7 Regina Maria Regional Laboratory, Medical Genetics Division, Regina Maria Private Health Network, Unirea Medical Center, 400363 Cluj-Napoca, Romania; anca.hadarean@reginamaria.ro; 8 Regina Maria Regional Laboratory, Laboratory Medicine Division, Regina Maria Private Health Network, Unirea Medical Center, 400363 Cluj-Napoca, Romania

**Keywords:** *Helicobacter pylori*, antibiotic resistance, ETEST^®^, GenoType HelicoDr, Romania

## Abstract

Little evidence has been published regarding the antimicrobial resistance patterns of *Helicobacter pylori* (*H. pylori*) strains in Northwestern and Central Romania. The aim of this study was to determine the antibiotic resistance pattern of *H. pylori* isolates from gastric biopsies collected from patients living in Romania using ETEST^®^ and GenoType HelicoDR. Gastric biopsies were obtained from 148 adult patients, 87 women and 61 men, the majority (131 patients) from Northwestern and Central Romania. Sixty-nine *H. pylori* strains were detected by both culture and PCR; sixty-three biopsies were negative by both techniques; one biopsy was positive by culture but negative by PCR; and fifteen biopsies were negative by culture but positive by PCR. Primary resistance against clarithromycin, fluoroquinolones, and metronidazole was found in 16.7%, 11.1%, and 13.3% of strains, respectively. No primary resistance has been detected against amoxicillin, tetracycline, and rifampicin. Secondary resistance against clarithromycin, fluoroquinolones, metronidazole, amoxicillin, tetracycline, and rifampicin was found in 75.8%, 30.3%, 65.5%, 1.8%, 1.8%, and 7.3% of the strains, respectively. The most frequent clarithromycin-resistant genotype detected by GenoType HelicoDR was A2147G (62.3%). Concordances between ETEST^®^ and PCR for clarithromycin and fluoroquinolones were 85.5% and 78.3%, respectively. Further investigation of *H. pylori* resistance should be conducted to ensure proper eradication schemes.

## 1. Introduction

*Helicobacter pylori* (*H. pylori*) is a Gram-negative bacillus involved in the development of gastritis, gastric and duodenal ulcers, gastric cancer, and MALT (mucosa-assisted lymphoid tissue) lymphoma. The eradication of *H. pylori* infection implies the use of inhibitors of gastric acid secretion and various combinations of antibiotics: macrolides, amoxicillin, metronidazole, quinolones, tetracyclines, and bismuth [1,2,3,4,5]. Knowing resistance patterns to antibiotics constitutes valuable information for an effective eradication treatment. The overuse of antibiotics has induced the selection of resistant strains in many bacterial species, leading to important challenges regarding the choice of an effective therapeutic strategy. In this respect, *H. pylori* is no exception; several studies reported an increase in its antibiotic resistance rate over time [1,2,3,4]. One of the key points of current eradication schemes is dealing with local resistance to clarithromycin (CH) [5,6]. In 2017, clarithromycin-resistant *H. pylori* was selected by the World Health Organization (WHO) as an area of high priority for antibiotic research and development [2].

In Romania, according to several studies [7,8], the prevalence of *H. pylori* infection was higher than 50%, but more recent evidence suggests that this prevalence may actually be at a lower level, following a descendent trend [9,10]. However, there is still very limited published data about *H. pylori* resistance to antibiotics in adults or pediatric patients in Romania [9,11,12]. Nevertheless, the available information about other drug-resistant bacteria (*Klebsiella*, *E. coli*, *Pseudomonas*, *Enterococcus*) suggests alarming resistance rates (https://atlas.ecdc.europa.eu/, accessed on 18 September 2023), not always resembling those found in the neighboring countries [13,14,15].

Regarding *H. pylori* antibiotic resistance in adult patients, several European studies were conducted, but Romania was not among the participating countries [1,4,16,17]. In a neighboring country, Bulgaria, primary resistance to clarithromycin in elderly patients was 22.6% [18,19]. These data may offer certain clues about the expected clarithromycin resistance in Romania, but systematic investigations of *H. pylori* resistance in this part of Europe are certainly required.

Detection of antibiotic resistance patterns can be performed using phenotypic and/or genotypic methods. By cultivating gastric biopsies, *H. pylori* cultures can be obtained and antibiotic susceptibility testing against several antibiotics used in eradication cures can be performed. ETEST^®^ (bioMérieux, Marcy-l’Etoile, France) is a recommended and commercially available method that allows the determination of the minimum inhibitory concentration (MIC) for each tested antibiotic [20,21]. The European Committee on Antimicrobial Susceptibility Testing (EUCAST) (www.eucast.org, accessed on 15 November 2023) provides and updates the interpretation criteria, based on which an *H. pylori* strain is classified as resistant or susceptible to a certain antibiotic.

Compared to the dilution methods, ETEST^®^ is an easier method to use in daily practice, and previous studies have suggested that it could be a reliable alternative method for testing *H. pylori* susceptibility to antibiotics [22,23].

In-house and commercial molecular tests are available for *H. pylori* DNA detection from biopsies and/or culture, along with the determination of genetic mutation conferring resistance to clarithromycin and/or fluoroquinolones [24,25]. Among commercially available tests, GenoType Helico DR (Hain Lifescience GmbH, Nehren, Germany) uses the DNA strip methodology and allows the detection of mutations predictive of clarithromycin and fluoroquinolone resistance in *H. pylori* with values of sensitivity and specificity above 85% [26,27,28,29]. In Romania, a team from the Southeastern part of the country investigated clarithromycin and fluoroquinolones resistance of *H. pylori* from gastric biopsies using this molecular method [12]. Variations in antibiotic resistance can be present inside the same country between different regions [20]. Therefore, knowing that the local antibiotic resistance pattern is important, determining it can offer very necessary guidance to clinicians for the choice of optimal treatments for *H. pylori* eradication.

Hence, the aims of this study were (i) to detect the antibiotic resistance pattern of *H. pylori* isolates from gastric biopsies collected from patients living in Northwestern and Central Romania using ETEST^®^ (bioMérieux, Marcy-l’Etoile, France) and a PCR method, GenoType HelicoDR (Hain Lifescience GmbH, Nehren, Germany), and (ii) to evaluate possible phenotypic/genotypic correlations of this pattern for clarithromycin and fluoroquinolones.

## 2. Results

Gastric biopsies were obtained from 148 patients, 87 women (58.8%) and 61 men (41.2%), with ages between 20 and 84 years. Most patients (88.5%) came from Cluj County and other counties situated in Northwestern and Central Romania (Alba, Bihor, Bistrița-Năsaud, Brașov, Hunedoara, Maramureș, Mureș, Satu-Mare, Sălaj, Sibiu). The majority of patients (82.1%) lived in urban settings.

Among the 148 gastric biopsies, 69 biopsies (46.6%, 95% CI 38.4–54.9%) were positive by culture and PCR; 63 biopsies were negative by both culture and PCR; one biopsy was positive by culture but negative by PCR; 15 biopsies were negative by culture and positive by PCR (Table 1).

The Positive Percent Agreement (PPA) and Negative Percent Agreement (NPA) of GenoType HelicoDR compared to *H. pylori* culture were PPA = 98.6% (95% CI 92.3–99.9%) and NPA = 80.8% (95% CI 70.3–88.8%), respectively.

Among the 85 patients with *H. pylori*-positive cultures and/or PCR, 18 were naïve patients, without previous eradication therapy, and 67 were previously treated patients who had already attempted one or several eradication cures.

### 2.1. Clarithromycin Resistance Pattern

For CH, among the eighteen *H. pylori* strains detected by PCR from patients without previous eradication therapy, fifteen strains, i.e., 83.3% (95% CI 57.7–95.6%) were wild type (WT), and primary resistance to CH was detected in three strains, i.e., 16.7% (95% CI 4.4–42.3%). All three *H. pylori* CH-resistant strains showed A2147G mutation (MUT3 profile).

**Figure 1:**
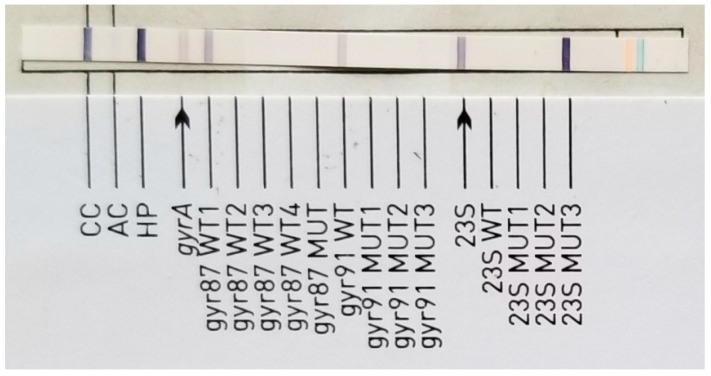
Clarithromycin (CH)-resistant *H. pylori* strain with 23S MUT3 profile (corresponding to A2147G mutation) determined by GenoType HelicoDR.

For CH, among the 66 *H. pylori* strains from PCR-positive biopsies with previous eradication cures, 16 (24.2%) strains were WT, corresponding to 24.2% (95% C 14.9–36.6%). Among the *H. pylori* strains which exhibited resistance to CH (50/66, 75.8%, 95% CI 63.4–85.1%), thirty-three strains exhibited a 23S MUT3 profile (corresponding to A2147G mutation), as exemplified in Figure 1, and seven strains showed 23S MUT1 profile (corresponding to A2146G mutation).

In 13 biopsies, a heterogenic population was observed, exhibiting a mixture of a resistant (23S MUT3) and a wild type sequence (Table 2).

Among the fifty CH-resistant *H. pylori* strains, thirty-eight strains were isolated from patients who received previous eradication cure with CH, and twelve CH-resistant strains and five CH susceptible strains were from patients who did not receive a previous cure with CH; *p* = 0.74–Fisher’s exact test; and RR = 1.1 (95% CI 0.78–1.55).

Overall, for CH, the concordance between ETEST^®^ and PCR can be summarized as follows: among the 69 *H. pylori* strains, 35 strains were resistant and 24 strains were susceptible by both ETEST^®^ and PCR; hence, there was a concordance of 59/69 strains, or 85.5% (95%CI 74.5–92.5%). The “discordant” remaining 10 strains were susceptible to ETEST^®^ (MIC 0.016–0.064 µg/mL) while still exhibiting resistance genes after PCR (Table 3).

### 2.2. Fluoroquinolones Resistance Pattern

For FQ, among the 18 *H. pylori* strains detected by PCR from naïve patients, 16 strains, i.e., 88.9% (95% CI 63.9–98.1%) were WT. Primary resistance to FQ was detected in 2 strains, i.e., 11.1% (95% CI 1.95–36.1%). (The two patients with *H. pylori* FQ-resistant strains were a 57-year-old female and a 67-year-old man.)

Interestingly, in the gastric biopsy of the patients with primary resistance to FQ, heterogeneity of the *H. pylori* population was noticed, with more than one hybridization band observed at one codon: a resistant and a wild type sequence (gyr87WT1 and gyr87MUT = N87K) and a mixture of two resistance sequences, respectively (gyr91MUT1 D91N and gyr91MUT2 D91G). One of the resistant *H. pylori* strains to FQ was also resistant to CH.

Among the 66 *H. pylori* strains from PCR-positive biopsies with previous eradication cure, 46 (69.7%, 95% CI 57–80.1%) were WT strains and 20 (30.3%, 95% CI 19.9–43%) were resistant to FQ, showing different resistant profiles: 13/20 showed heterogeneity of population, a resistant and a wild type sequence (Table 4). Among the 20 *H. pylori* FQ-resistant strains, 14/20 (70%) were isolated from women aged between 33 and 75 years old.

Among the twenty FQ-resistant *H. pylori* strains, fifteen strains were isolated from patients who received previous eradication cure with FQ, and five FQ-resistant strains and thirty FQ-susceptible strains were from patients who did not receive a previous cure with FQ, suggesting the existence of a link between previous treatment with FQ and *H. pylori* resistance to FQ: χ^2^(1, N = 66) = 9.1, *p* < 0.01; risk ratio RR = 3.4 (95% CI 1.4–8.2); and risk difference RD = 34.1% (95% CI 13–55.2%).

Overall, for FQ, the concordance between ETEST^®^ and PCR can be summarized as follows: among the 69 *H. pylori* strains, 15 strains were resistant and 39 strains were susceptible by both ETEST^®^ and PCR, a concordance of 54/69 strains, or 78.3% (95% CI 66.4–86.9%). Five strains were susceptible by ETEST^®^ (MIC 0.19–0.38 µg/mL) but exhibited resistance genes by PCR; ten strains were resistant by ETEST^®^ (MIC 4–32 µg/mL) but exhibited no resistance genes by PCR (Table 5).

### 2.3. Amoxicillin, Tetracycline, Metronidazole, and Rifampicin Resistance Pattern

**Figure 2:**
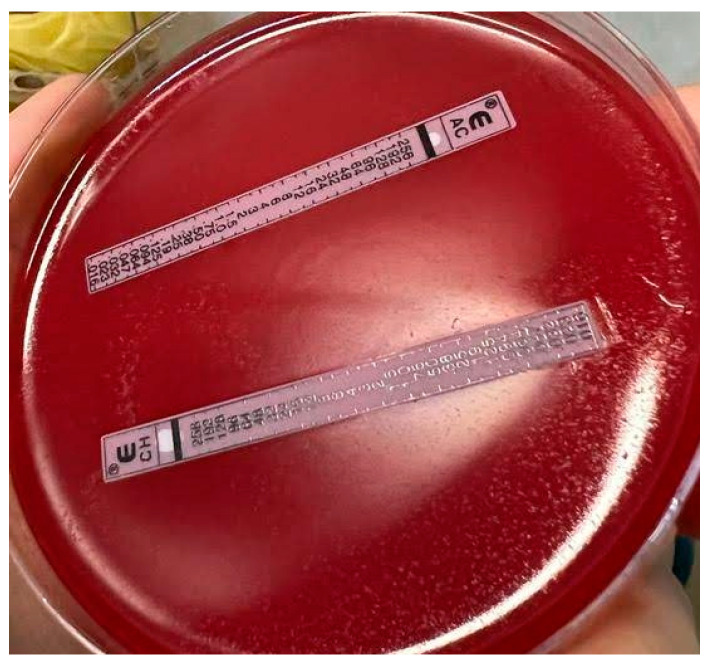
Amoxicillin and clarithromycin susceptibility determined by ETEST^®^ in one of the *H. pylori* strains isolated from naïve patients.

Among naïve patients, all fifteen isolated strains were susceptible to amoxicillin (MIC 0.016–0.032 µg/mL), tetracycline (MIC 0.032–0.125 µg/mL), and rifampicin (MIC 0.064–0.5 µg/mL); two *H. pylori* strains were resistant to metronidazole (2/15 strains, 13.3%, 95% CI 2.3–41.6%), with MIC 256 µg/mL. Figure 2 exemplifies antibiotic susceptibility testing performed by ETEST^®^ on one of the *H. pylori* strains isolated from naïve patients.

Among the fifty-five *H. pylori* strains isolated from patients who received previous antibiotic treatment, one strain was resistant to tetracycline (1/55, 1.8%) with MIC 4 µg/mL. Four strains were resistant to rifampicin (4/55, 7.3%) with MIC 1.5–32 µg/mL. Regarding metronidazole, 19 *H. pylori* strains were susceptible (19/55 strains, 34.6%, 95% CI 22.6–48.7%), with MIC 0.016–4 µg/mL, and 36 strains were resistant (36/55 strains, 65.45%, 95% CI 51.3–77.4%), with MIC 24–256 µg/mL (half of them, 18/36, were isolated from patients who received a previous eradication cure with metronidazole).

We detected only one *H. pylori* strain resistant to amoxicillin (1/55, 1.8%), with MIC 256 µg/mL, isolated from a 20-year-old female patient who received a previous eradication cure with AMX and CH (Table 6). This strain also showed resistance to CH, with MIC 256 µg/mL and a 23S MUT3 profile (corresponding to A2147G mutation), as well as resistance to rifampicin (MIC 32 µg/mL).

### 2.4. Overall Antibiotic Resistance Pattern

The overall antibiotic resistance profile of the *H. pylori* strains detected by culture and PCR is presented in Table 6.

Multidrug resistance (MDR, simultaneous resistance to ≥3 antibiotics of different classes) was found in 17 *H. pylori* strains (20%), almost all of them (15 strains) being resistant to CH+FQ+MET. The MDR *H. pylori* strain exhibiting resistance to CH+MET+RIF (Table 6) was isolated from a 41-year-old female patient who had received a previous eradication cure with FQ and MET. This strain exhibited a 23S MUT3 profile (corresponding to A2147G mutation) and the following MIC pattern: 16 µg/mL to CH, 1.5 µg/mL to RIF, and 256 µg/mL to MET. Among the fifteen strains resistant to CH+FQ+MET, nine were isolated from female patients (32–75 years old) and six were isolated from male patients (30–53 years old); all of these patients had received previous eradication cure, as shown in Table 7.

## 3. Discussion

*Helicobacter pylori* is a bacterium with special growth requirements [30]. Therefore, its cultivation is difficult (sometimes with reported sensitivity values below 70%) and time-consuming (up to two weeks, including antimicrobial susceptibility testing) [20]. Thus, not all microbiology laboratories are able to provide high-quality results. Multiple factors can influence the successful cultivation of *H. pylori* from gastric biopsies: the number and site of biopsies, recent (2–4 weeks) treatment with antibiotics and/or proton pump inhibitors, means of storage and transportation time of biopsies to the laboratory, proper processing of biopsy specimens, culture media, and atmosphere during incubation, and even the possible contamination of biopsies with urease, producing bacterial species [30,31].

*Helicobacter pylori* detection by culture and antibiotic susceptibility testing is fastidious and, due to numerous difficulties, scarcely performed in Europe [16,32]. The main advantage of cultivating *H. pylori* is that antibiotic susceptibility testing to antibiotics, as part of different treatment strategies, can be performed afterward.

It has been demonstrated that compared to real-time PCR from gastric biopsies, culture has a lower sensitivity [24,33,34]. Among commercial tests, GenoType HelicoDR (Hain Lifescience GmbH, Nehren, Germany) uses the DNA strip methodology and allows the detection of mutations predictive of clarithromycin and fluoroquinolone resistance in *H. pylori* [26,27,28,29]. Similar to other reports, the PCR method used in this study was able to detect *H. pylori* in more gastric biopsies compared to culture. In our study, the Positive Percent Agreement (PPA) and Negative Percent Agreement (NPA) of GenoType HelicoDR compared to *H. pylori* culture were PPA = 98.6% (95% CI 92.3–99.9%) and NPA = 80.8% (95% CI 70.3–88.8%), respectively.

Concerning *H. pylori* resistance to CH, for the 69 strains detected by both culture and PCR in our study, concordance between ETEST^®^ and PCR was 85.5% (95% CI 74.5–92.5%). GenoType HelicoDR also allowed the detection of heterogeneous strains in our study; a mixture of a resistant and a wild type sequence detected in 13 biopsies (15.5%). The majority of CH-resistant genotypes detected by GenoType HelicoDR were A2147G (33/53 CH-resistant strains, 62.3%). This mutation was found as the main mutation in several previous studies, including in the study in Southeastern Romania [12,26,27,29].

Overall, regarding resistance to FQ, in our study, we obtained a concordance between ETEST^®^ and PCR in 54/69 strains, or 78.3% (95% CI 66.4–86.9%). The majority (15/22, 68.2%) of FQ-resistant strains detected by GenoType HelicoDR showed a heterogeneity of population, with more than one hybridization band observed at one codon: a resistant and a wild type sequence. The main detected mutations were N87K, D91G, and D91N. In the study in Southeastern Romania, primary FQ was detected in 30% of patients (27/90), and the most common mutations were D91N, D91G, and N87K [12].

The presence of a dual population (heteroresistance), both susceptible and resistant at the same time in the gastric biopsies of the same patient, is not uncommon [24,26,27,28,29,34,35]. Dual populations can be found in both naïve patients and those previously exposed to eradication treatment. Dual populations were also found in 15.2% of patients when antibiotic susceptibility was studied separately for antrum and corpus biopsies [36]; in these cases, DNA fingerprinting analysis suggested infection with a single strain of *H. pylori* since no substantial differences among DNA patterns were found. The authors concluded that different antibiotic susceptibility between antrum and corpus biopsies could be a possible explanation for treatment failure [36].

One of the advantages of PCR methods from gastric biopsies is that they do not necessitate special transport conditions, in contrast to culture; however, a biopsy requires an invasive harvesting method. During recent years, DNA extraction kits from stool samples were developed and improved, with sensitivity and specificity above 90%. Consequently, PCR on stool samples is possible and it could represent a non-invasive alternative to PCR from gastric biopsies [6,37].

Data about *H. pylori* antibiotic resistance in Europe are known [1,2,4,16,17], but only scarce data in Romania are available [11,12], with eradication treatments being empirical in most cases, yielding significant risks for infection relapse. As emphasized in the last Maastricht VI/Florence consensus report, **clarithromycin** is currently a key antibiotic to eradicate *H. pylori*, but when resistance is present, the probability of treatment success is low [6]. The threshold of 15% clarithromycin resistance previously proposed [5] was exceeded in many countries [2,19]. These findings advocate for regular testing of clarithromycin resistance.

In the current study, primary resistance to CH was found in 16.7% (95% CI 4.4–42.3%) of the strains, but the number of naïve patients was quite low (only 18 patients), so further investigation of primary *H. pylori* resistance in this part of Romania will be necessary. Secondary resistance of *H. pylori* to CH was 75.8% (95% CI 63.4–85.1%). No significant difference was found between the proportion of CH-resistant strains isolated from patients who received previous eradication cures with CH and those who were never treated with CH (*p* = 0.74—Fisher’s exact test, RR = 1.1, 95% CI 0.78–1.6).

In Europe, *H. pylori* resistance to antibiotics varies according to country. Several European studies presented differences in resistance between different parts of the continent, with significantly higher resistance in Central/Western and Southern Europe compared to Northern European countries [16,35]. In naïve patients, resistance in Southern Europe (Italy, Spain, Greece) was higher than 20% for clarithromycin, as opposed to Norway with rates below 10% [16]. In the same study, clarithromycin resistance following a first eradication treatment attempt was higher than 60% [16]. Primary and secondary resistance to clarithromycin in France were 20.9% (16.3–26.4) and 56.4%, respectively [38].

In a 2018 systematic review and meta-analysis [2], the pooled prevalence of *H. pylori* secondary clarithromycin resistance in Europe was 48% (95% CI, 38%–57%); it was also suggested that the development of resistance was associated with an increased risk of treatment failure, and the OR for failure of clarithromycin-containing regimens was 6.97 (95% CI 5.23 to 9.28).

In our study, secondary resistance to **fluoroquinolones** was found in 30.3% 95% CI 19.9–43% (20/66 strains). Among the twenty FQ-resistant *H. pylori* strains, fifteen strains were isolated from patients who received previous eradication cure with FQ; five FQ-resistant strains and thirty FQ-susceptible strains were from patients who did not receive a previous cure with FQ, suggesting the existence of a link between previous treatment with FQ and *H. pylori* resistance to FQ: χ^2^(1, N = 66) = 9.1, *p* < 0.01, RR = 3.4 (95% CI 1.4–8.2), and RD = 34.1% (95% CI 13–55.2%).

For FQ, primary resistance was detected in two gastric biopsies, representing 11.1% (95% CI 1.95–36.1%) of *H. pylori* strains. In these gastric biopsies, heterogeneity of the *H. pylori* population was observed, with more than one hybridization band observed at one codon. But, as stated above, the number of naïve patients was quite low in our study (only 18 patients), so further studies will need to complete the investigation of primary resistance in this part of Europe.

Like in the case of clarithromycin, in naïve patients, resistance in Southern Europe (Italy, Spain, Greece) was higher than 20% for levofloxacin, as opposed to Norway with rates below 10%; levofloxacin resistance after a first eradication treatment was 28% [16]. In France, primary resistance to levofloxacin was 17.6% and secondary resistance was 22.7%, respectively [38].

High levofloxacin resistance rates (>30%) were found in untreated Italian adults (33.8% in 2015–2019) and in consecutive Bulgarian patients (30.6% in 2016–2022) [19,39].

Several studies showed significant associations between *H. pylori* clarithromycin resistance and the consumption of macrolides in the community and between levofloxacin resistance and consumption of quinolones, confirming the positive correlation between macrolide and quinolone consumption in the community and corresponding *H. pylori* resistance in European countries. For this reason, it was suggested that *H. pylori* treatment with clarithromycin and levofloxacin should not be started without susceptibility testing in most European countries [2,4,17,40].

Consumption of antibacterials for systemic use in Romania, as recorded for 2021 by the European Centre for Disease Prevention and Control (ECDC), was the highest in Europe: 24.3 DDD (defined daily dose)/1000 inhabitants/day. The specific consumption of quinolones was recorded at 3.2 DDD/1000 inhabitants/day and 4.7 DDD/1000 inhabitants/day for macrolides, both constituting the second highest levels in Europe (after Bulgaria) for these particular antibacterial agents [41].

A very recent review regarding the evolution of antibiotic resistance in *H. pylori* over the years [19] showed that, fortunately, the prevalence of *H. pylori* resistance to **amoxicillin** appears to remain low in most countries; it was less than 2% in most European countries [2,17,33]. Despite this low amoxicillin resistance rate, in Bulgaria, a significant increase in overall amoxicillin resistance was detected in consecutive Bulgarian patients (a 2.1-fold rise from 2007 to 2014 until 2015–2021) [19].

In the current study, we detected only one *H. pylori* strain resistant to amoxicillin: a strain that had been isolated from a patient who received a previous eradication cure with AMX and CH.

**Metronidazole** could be an antibiotic of choice for infections caused by intestinal (e.g., *Giardia*) or vaginal protozoa (*Trichomonas vaginalis*) and anaerobic bacteria (like *Clostridioides difficile*), justifying its use in various infections (parasitic, gastrointestinal, gynecological, etc.). Precisely, this use of metronidazole can contribute to an increase in *H. pylori* resistance to this antibiotic [35].

In our study, secondary resistance to metronidazole was 65.5%, 95% CI 51.3–77.4% (36/55 strains), with MIC 24–256 µg/mL (half of them, 18/36, were isolated from patients who received previous eradication cure with metronidazole). This elevated resistance rate is consistent with other reports. In France, for instance, primary resistance to metronidazole was 58.6% and secondary resistance was 87.3%; in Poland, primary resistance was 42%; in Italy, primary resistance was 38.6%; in Bulgaria, primary resistance was 49.7% [2,3,19,38,42]. However, we must take into consideration that for metronidazole resistance determined by ETEST^®^, several articles described elevated MICs for metronidazole or errors compared to the dilution method [21,22,23,43,44].

In our study, only one strain of seventy isolated *H. pylori* strains was resistant to tetracycline: a strain isolated from a patient that previously received several antibiotic cures. In a systematic review and meta-analysis published in 2018, during 2006–2016, a very low tetracycline resistance was found [2]. In France, the results of the *H. pylori* resistance survey during 2018, as well as the evolution over a previous 5-year period, found no resistance to tetracycline and a very low resistance to rifampin (1.2%) in both naïve and treated patients [38].

Only four *H. pylori* strains (4/70) were resistant to rifampicin in our study, which is good news in a country where tuberculosis remains a serious public health problem [45]. According to a recent study, after one or more failures of eradication treatment, rifabutin-containing therapy represents an effective and safe strategy [46].

Recent reports have indicated the alarming prevalence of multidrug-resistant (MDR) strains with triple resistance to clarithromycin, metronidazole, and quinolones [19,40,47]. In our study, among the 85 strains detected by culture and/or PCR, **single-drug resistance** was detected in 19 *H. pylori* strains (22.3%), and the majority of them (16 strains) were resistant to CH. **Dual resistance** was found in 25 *H. pylori* strains (29.4%), with more than half resistant to CH+MET. **Triple resistance** (multidrug resistance) was found in 17 *H. pylori* strains (20%), and almost all of them (15 strains) were resistant to CH+FQ+MET.

In a time trend analysis of *H. pylori* resistance to antibiotics in Europe published in 2021, dual clarithromycin and metronidazole resistance was higher than 10% during most of the study period, 2013–2020 (except in 2020, 7.3%). Triple clarithromycin, metronidazole, and levofloxacin resistance were higher than 5%, except in 2019 (4.1%) and 2020 (0.9%). After the failure of the first eradication treatment, dual and triple resistances were found in 43% and 19% of patients, respectively. After the failure of four eradication treatments, dual and triple resistances increased to 63% and 39%, respectively [16]. In another study, dual resistance to both clarithromycin and metronidazole was reported in 46% of the cases, and triple resistance to clarithromycin, metronidazole, and levofloxacin in 39% [46].

Contingent with the local budget, a strategy for *H. pylori* detection should be applied. Knowing the local resistance pattern is important; it can offer guidance to clinicians in choosing the best treatment to obtain eradication. Even though the number of tested *H. pylori* strains in this study was small compared to other European studies, the obtained data may still shed some light on the antibiotic resistance pattern of *H. pylori* in this part of Eastern Europe.

## 4. Materials and Methods

Between May 2021 and August 2023, gastric biopsies were collected as much as possible from consecutive patients (contingent on patient consent and clinical indication of a biopsy) who presented symptoms suggestive of *H. pylori*-related gastric disease at the gastroenterology departments of 3 university clinics in Cluj-Napoca, Romania, which treat patients mainly from Northwestern and Central Romania.

Additional patient biopsies were collected from 2 private clinics in Cluj-Napoca, Romania, 2 clinics in Oradea, and 1 clinic in Baia-Mare (both in Northwestern Romania), as well as 1 clinic in Brașov (in Central Romania).

Both *H. pylori* eradication-naïve patients and patients with a previous *H. pylori* eradication attempt were included in the present study. The decision to perform a gastroscopy and collect gastric biopsies was inevitably influenced by clinical correlations of the treating gastroenterologists.

Exclusion criteria were applied as follows: patients younger than 18 years, delays in the transport of biopsies by 48 hours or more, and treatment with antibiotics and/or proton pump inhibitors (PPIs) administered 15 days before the endoscopy or less (all collaborating gastroenterologists were instructed to interrupt antibiotic treatment at least one month prior to harvesting a gastric biopsy for this study, but this was sometimes inadvertently omitted; therefore, this last exclusion criterion has been reinforced instead in order to ensure an acceptable recruitment level and sample size).

This study was conducted according to the 2013 revision of the Helsinki Declaration (Fortaleza) and was approved by the research ethics committee of the Iuliu Hatieganu University of Medicine and Pharmacy, Cluj-Napoca, Romania (No. 163/2.04.2018 and No. 41/18 February 2022).

Gastric biopsies were collected from all consenting patients during gastroscopy from both the antrum and the body of the stomach. The biopsies were placed together in a Portagerm pylori container (bioMérieux, Marcy-l’Etoile, France) and transported to the laboratory of the Regina Maria Laboratory Medicine Division, Unirea Medical Center, Cluj-Napoca, Romania. There, the biopsies were ground together in 1 mL of broth using a manual homogenizer, according to standard procedures described in the literature [33,34]. Afterward, each sample was divided in two, one for bacterial culture (cultivated immediately) and the other for a molecular assay (stored at −80 °C until processed).

A part of the suspension was inoculated immediately after arriving in the laboratory on a selective medium, Pylori Agar (bioMérieux, Marcy-l’Etoile, France) for cultivation [30]. During incubation at 37 °C for a total of 14 days, a microaerophilic atmosphere was obtained using CampyGen^TM^ Compact sachets (Oxoid Ltd., Basingstoke, UK). The sachets were changed, and the culture media were checked for the presence of *H. pylori* colonies every other day. The colonies resembling *H. pylori* were identified by positive oxidase, catalase, urease tests, and microscopy.

For antibiotic susceptibility testing (AST), a fastidious Mueller–Hinton agar medium (MH-F) supplemented with 5% defibrinated horse blood and 20 mg beta-NAD (bioMérieux, Marcy-l’Etoile, France) was used. The inoculum was prepared from a 2-day-old subculture and was adjusted to an opacity equivalent of 3 McFarlands (approx. 10^8^ colony forming units, CFU/mL). The AST results were read after 48–72 h of incubation at 37 °C in a microaerophilic atmosphere, provided that growth was clearly visible. The MIC values of the antibiotics, amoxicillin (AMX), clarithromycin (CH), levofloxacin (FQ), metronidazole (MET), rifampicin (RIF), and tetracycline (TET) tested by ETEST^®^ (bioMérieux, Marcy-l’Etoile, France) were read where the inhibition ellipse intersects the strip. Interpretive criteria for susceptibility vs. resistance after ETEST^®^ were chosen according to the *European Committee on Antimicrobial Susceptibility Testing Breakpoint Tables* for the interpretation of MICs and zone diameters, Version 13.0, 2023, valid from 1 January 2023 (confirmed by Version 13.1, 2023, valid from 29 June 2023) [48]. The *H. pylori* strain CCUG 17,874 was used for quality control.

The method used for *H. pylori* DNA detection from biopsies and the detection of clarithromycin and fluoroquinolones resistance was GenoType HelicoDR (Hain Lifescience GmbH, Nehren, Germany). The test uses the DNA strip methodology and allows the detection of mutations predictive of clarithromycin and fluoroquinolone resistance in *H. pylori*. The probes are designed to hybridize with the sequences of the wild type alleles (WT probes) or the mutated alleles (MUT probes) [25]. GenoType HelicoDR can identify mutations in A2146G, A2146C, and A2147G for 23S RNA (encoding clarithromycin resistance) and in N87K, D91N, D91G, and D91Y for the *gyr*A gene (encoding fluoroquinolones resistance) [12,26,27,28,29].

For molecular assays, the parts of the ground biopsies frozen at −80 °C were used. According to the manufacturer’s instructions, 3 steps were performed: DNA extraction from gastric biopsies, multiplex amplification, and hybridization.

For DNA extraction from gastric biopsies, after overnight digestion with proteinase K at 56 °C, DNA was manually extracted using QIAamp^®^ DNA Mini Kit (QIAGEN GmbH, Hilden, Germany) while observing the manufacturer’s instructions.

For multiplex amplifications with biotinylated primers, according to the manufacturer’s instructions, we used the following ingredients for each sample: 35 µL primer nucleotide mix, 5 µL 10× polymerase buffer, 2 µL 25 mM MgCl_2_ solution, 0.2 µL HotStarTaqDNA Polymerase (QIAGEN GmbH, Hilden, Germany), 3 µL water, and 5 µL DNA. Samples were then incubated in a thermal cycler Applied Biosystems™ProFlex™ PCR System, 2 × 96 wells (Thermo Fisher Scientific Inc., Waltham, MA, USA), with the following parameters: 1 cycle for 15 min at 95 °C; 10 cycles at 95 °C for 30 s and 2 min at 58 °C; 25 cycles at 95 °C for 25 s, plus 40 s at 53 °C, plus 40 s at 70 °C; and 1 cycle for 8 min at 70 °C.

Hybridization included several steps: chemical denaturation of the amplification products (20 µL for each sample), hybridization of the single-stranded, biotin-labeled amplicons to membrane-bound probes, stringent washing, the addition of a streptavidin/alkaline phosphatase conjugate, and an alkaline phosphatase mediated staining reaction (manufacturer’s instructions).

For the evaluation and interpretation of the results, the positive bands on DNA strips were analyzed using the provided template for aligning the observed bands with the respective locus control bands.

Data have been collected and described using Microsoft Excel 2010. Confidence intervals for proportions have been computed using VassarStats (VassarStats: Website for Statistical Computation. Available from http://www.vassarstats.net, accessed on 17 October 2023), with continuity corrections based on a method described by Robert Newcombe [49].

Positive Percent Agreement (PPA) and Negative Percent Agreement (NPA) have been used as diagnostic indicators (computed similarly to sensitivity and specificity) since both culture-based and PCR-based methods are subject to a degree of uncertainty, and neither of them taken separately could be considered a diagnostic gold standard for detecting *H. pylori* infection. Confidence intervals for diagnostic indicators have been computed using the MedCalc online statistical calculator (MedCalc Software Ltd., Ostend, Belgium. Available from https://www.medcalc.org/calc/diagnostic_test.php, accessed on 20 October 2023).

For the existence of a significant link between previous treatments with CH or FQ and the selection of *H. pylori* strains resistant to CH, respectively, FQ has been investigated using chi-square or Fisher’s exact tests (depending on the size of expected frequencies). The risk ratio (RR) and risk difference (RD) for those suspected links have also been computed along with their 95% CI using Epi Info™ (Centers for Disease Control and Prevention, Atlanta, GA, USA).

## 5. Conclusions

Knowing local patterns and trends of *H. pylori* antibiotic resistance, as well as previous antibiotic use, is essential for obtaining high eradication rates. In our study, primary resistance to clarithromycin, fluoroquinolones, and metronidazole was found, respectively, in 16.7% (95% CI 4.4–42.3%), 11.1% (95% CI 1.95–36.1%), and 13.3%, 95% CI 2.3–41.6%) of the investigated strains. We also detected high secondary resistance rates to clarithromycin, fluoroquinolones, and metronidazol of 75.8%, 30.3%, and 65.5%, respectively. Fortunately, no primary resistance against amoxicillin, tetracycline, and rifampicin has been detected, and secondary resistance rates against these antibiotics were at low levels: 1.8%, 1.8%, and 7.3%, respectively. Our results suggest that further investigation of *H. pylori* resistance could be an important step in order to ensure proper eradication schemes. Even though the number of *H. pylori* strains tested in this study was relatively small, the obtained results shed some light on the antibiotic resistance pattern of *H. pylori* in Northwestern and Central Romania. The current study also suggests that further investigation of *H. pylori* resistance and the development of a regional *H. pylori* identification and resistance surveillance center could benefit current and future patients while also limiting the acquisition and spread of antibiotic resistance among circulating *H. pylori* strains from this part of Europe.

## Figures and Tables

**Table 1:** Results of *Helicobacter pylori* (*H. pylori*) detection by culture and by GenoType HelicoDR.

		Culture		
		Positive	Negative	Total
Genotype HelicoDR	positive	69	15	84
	negative	1	63	64
Total		70	78	148

**Table 2:** Genotypes of *H. pylori* strains detected by GenoType HelicoDR for clarithromycin.

Genotype	No. of Strains (%)
WT (wild type)	31 (36.9%)
23S MUT1, A2146G mutation	7 (8.3%)
23S MUT2, A2146C mutation	0
23S MUT3, A21467 mutation	33 (39.3%)
WT + 23S MUT3 A21467 mutation	13 (15.5%)
Total	84 (100%)

**Table 3:** Clarithromycin susceptible and resistant *H. pylori* strains detected by culture and GenoType HelicoDR.

Clarithromycin		Genotype HelicoDR		
		Wild Type	Resistance Genes	Total
ETEST^®^	susceptible	24	10	34
	resistant	0	35	35
Total		24	45	69

**Table 4:** Genotypes of *H. pylori* strains detected by GenoType HelicoDR for fluoroquinolones.

Genotype	No. of Strains	%
gyr 87 WT 1–4	44 (28WT1+16WT2)	52.4%
gyr 87 WT1+WT2	1	1.2%
gyr 87 MUT, N87K	10	11.9%
gyr 91 WT	17	20.2%
gyr 91 MUT1, D91N	4	4.8%
gyr 91 MUT2, D91G	6	7.1%
gyr 91 MUT3, D91Y	1	1.2%
gyr91 MUT1+MUT2	1	1.2%
Total	84	100%

**Table 5:** Fluoroquinolones susceptible and resistant *H. pylori* strains detected by culture and GenoType HelicoDR.

Fluoroquinolones		Genotype HelicoDR		
		Wild Type	Resistance Genes	Total
ETEST^®^	susceptible	39	5	44
	resistant	10	15	25
Total		49	20	69

**Table 6:** Overall antibiotic resistance profile of *H. pylori* strains.

Antibiotics		*H. pylori* Strains from Patients with Previous Antibiotic Cure	*H. pylori* Strains from Naïve Patients	Total
*H. pylori* strains susceptible to all antibiotics		11	13	24 (28.2%)
*H. pylori* strains resistant to one antibiotic	CH	14	2	16 (18.8%)
	FQ	1	0	1 (1.2%)
	MET	1	1	2 (2.4%)
*H. pylori* strains resistant to two antibiotics	CH+FQ	4	1	5 (5.8%)
	CH+MET	15		15 (17.6%)
	MET+RIF	2		2 (2.4%)
	MET+TET	1		1 (1.2%)
	MET+FQ	1	1	2 (2.4%)
*H. pylori* strains resistant to three antibiotics	CH+FQ+MET	15		15 (17.6%)
	AMX+CH+RIF	1		1 (1.2%)
	CH+MET+RIF	1		1 (1.2%)
Total		67	18	85 (100%)

CH = clarithromycin, FQ = fluoroquinolone; MET = metronidazole; AMX = amoxicillin; TET = tetracycline; RIF = rifampicin.

**Table 7:** The fifteen MDR *H. pylori* strains resistant to CH+FQ+MET.

Sample No.	Sex	Age (Years)	CH Type of Mutation	FQ Type of Mutation	Antibiotics Used in Previous Eradication Cure
8	M	45	A2147G	N87K	AMX+FQ
10	M	48	A2147G	N87K	AMX+CH
12	M	30	A2147G	N87K	AMX+CH+FQ
17	M	36	A2146G	N87K	AMX+FQ+MET
25	M	53	A2147G	D91G	AMX+CH+FQ+MET
26	M	39	A2147G	D91Y	FQ+MET
2	F	57	A2147G	D91N	AMX+CH+FQ+MET+ TET
18	F	66	A2147G	D91G	CH+MET
30	F	41	A2147G	N87K	AMX+CH+FQ+MET+TET
43	F	32	A2147G	D91G	AMX+CH
68	F	37	A2147G	D91N	AMX+CH+FQ+TET
84	F	34	A2147G	D91N	AMX+FQ
122	F	75	A2147G	N87K	AMX+CH+FQ+MET+TET
126	F	44	A2147G	D91N	AMX+CH+FQ
132	F	52	A2147G	N87K	AMX+CH+FQ+MET

CH = clarithromycin, FQ = fluoroquinolone; MET = metronidazole; AMX = amoxicillin; TET = tetracycline; RIF = rifampicin; M = male; F = female

## Data Availability

Research data can be made available upon reasonable request addressed to the corresponding author.
